# Cost-effective handoff scheme based on mobility-aware dual pointer forwarding in proxy mobile IPv6 networks

**DOI:** 10.1186/2193-1801-3-57

**Published:** 2014-01-28

**Authors:** Seungsik Son, Jongpil Jeong

**Affiliations:** College of Information and Communication Engineering, Sungkyunkwan University, Suwon, Republic of Korea

**Keywords:** PMIPv6, Dual pointer forwarding, mDPF, mPF, Mobility management

## Abstract

In this paper, a mobility-aware Dual Pointer Forwarding scheme (mDPF) is applied in Proxy Mobile IPv6 (PMIPv6) networks. The movement of a Mobile Node (MN) is classified as intra-domain and inter-domain handoff. When the MN moves, this scheme can reduce the high signaling overhead for intra-handoff/inter-handoff, because the Local Mobility Anchor (LMA) and Mobile Access Gateway (MAG) are connected by pointer chains. In other words, a handoff is aware of low mobility between the previously attached MAG (pMAG) and newly attached MAG (nMAG), and another handoff between the previously attached LMA (pLMA) and newly attached LMA (nLMA) is aware of high mobility. Based on these mobility-aware binding updates, the overhead of the packet delivery can be reduced. Also, we analyse the binding update cost and packet delivery cost for route optimization, based on the mathematical analytic model. Analytical results show that our mDPF outperforms the PMIPv6 and the other pointer forwarding schemes, in terms of reducing the total cost of signaling.

## Introduction

In the present Internet environment, Mobile IPv6 (MIPv6) was proposed to support IP mobility, in accordance with developing broadband wireless network technology and mobile terminals (Johnson et al. 2004). However, signaling procedures are required to support the mobility of mobile terminals based on Mobile Node (MN) in MIPv6, and incur a higher signaling overhead on the network. The Internet Engineering Task Force (IETF) proposed Fast handover for MIPv6 (FMIPv6) (Koodli 2005) and Hierarchical Mobile IPv6 (HMIPv6) (Soliman et al. 2008) to eliminate the weaknesses of MIPv6, but the waste of wireless link resources and handover delay problem was not solved. Therefore, the Proxy Mobile IPv6 (PMIPv6) standard was established to solve the remaining problems (Gundavelli et al. 2008). The core of PMIPv6, in comparison with MIPv6 environment mobility management working in MN, is that mobility management is handled by newly introduced equipment in the network area. This means that the default IPv6 specification can get mobility service anytime, while the existing MIPv6 can get the same service, when the MN has complicated specification. Therefore MN, located in the PMIPv6 domain, can get the mobility services by network equipment using only the IPv6 specification.

**Figure 1:**
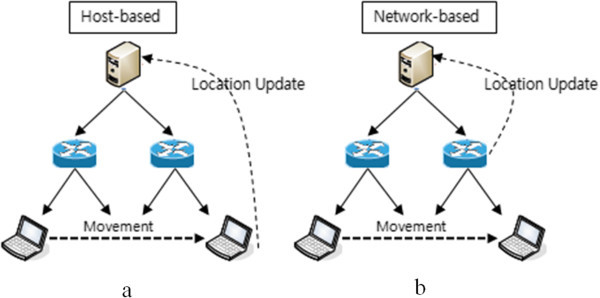
**The difference between (a) host-based, and (b) network-based mobility management.**

PMIPv6 proposed to solve the signaling overhead as a problem of mobility support, for MN-based has no different mobility management of the host-based (see the Figure 1). The only difference is that there is no request regarding mobility of the MN. In the case of host-based, when the MN moves within a Mobility Anchor Point (MAP), MN requires a binding update to the Home Agent (HA). And through it, the subnet address Regional Care-of-Address (RCoA) of MAP and the prefix address on-Link CoA (LCoA) of the Access Router are renewed, to establish the connection to the MN. However, in the case of PMIPv6, the network is in charge of this part, and the Mobility Access Gateway (MAG) recognizes the mobility of the L2 connection information, and registers the Proxy Binding Update (PBU) at a Local Mobility Anchor (LMA). LMA plays the role of HA to manage the MN being registered to the domain. In addition, LMA sends a Proxy Binding Acknowledgement (PBA), including a Home Network Prefix (HNP) for the MN, and the MN, connected to MAG, is connected by configuring the Home of Proxy Address (pHoA) based on the HNP. But, PMIPv6 resolves the domain internal signaling overhead, while the cross-domain is not specified for the handover. The inter domain handover in PMIPv6 is handover between the LMAs. Because LMA, one of the PMIPv6 domains, has the function of HA, Binding Update (BU) sends the HNP from pLMA to nLMA to carry out, and nLMA access to correspondent node (CN) with the information HNP received from pLMA. However, the binding update occurs when moving between the LMAs, and this is the cause of the high signaling overhead and handover delay.

Because it is an important issue in a large-scale mobile network to reduce the handoff delay and high signaling overhead, extensive research was conducted. The most typical way is the Pointer Forwarding (PF) scheme. The pointer forwarding techniques of the basic cellular network environment can be summarized as follows: When the MN moves, that domain is registered in the Visitor Location Register (VLR), and is connected by a forwarding pointer to the previous VLR and the Home Location Register (HLR), and the new location is not reported. These procedures will greatly reduce the signaling overhead of the HLR. The pointer frowarding is that the MN moves to multiple VLR domains in the form of chain links. When one session arrives for the first time, the MN that is located on that cellular network performs the initialization of the VLR (the starting point of the forwarding chain). MN refers to a pointer to the current VLR. Therefore, in order to limit the excessive delay on the location of the MN, the number of pointer chains can be extended, as long as the value of K, a predefined number. In other words, when the length of the pointer chain reaches K, it cannot be allowed to add a pointer, and should be registered in the HLR.

In this paper, a method is provided for improving the performance via a dual pointer transfer technique, for recognizing the movement by the PMIPv6 network environment. In the case of inter-domain movement in PMIPv6, a binding update is performed among pLMA, nLMA, and CN (LMA). That is, binding updates occur in which HNP is passed to nLMA in pLMA, and nLMA connects to CN (LMA). However, these binding update techniques will have high signal overhead and delay of handover. To solve those problems, the pointer chain is connected, when handover between pLMA and nLMA occurs.

When MN accesses nLMA, nLMA requests the address of HNP and pLMA of pLMA, and a pointer chain is formed between pLMA and nLMA, by transferring the data packet through the LBU from pLMA to nLMA. This enhanced technique can reduce the signal overhead and delay of handover, when an MN moves between domains. Furthermore, the mobile-aware pointer forwarding technique recognizes that mobility adaptive pointer forwarding scheme (mPF) is applied. Mobility aware reduces the negative factor of pointer forwarding in the packet transfer process of MN. In mPF, the slow or fast moving speed of the MN is recognized. When the moving speed is faster than a pre-defined stay time, the length of chain extends to K, while the negative factor of pointer forwarding is reduced, by notifying of CN (LMA) from MN, when the speed of MN is slow, and the packet is transferred. In addition, a mathematical analytic model is used to calculate the updating of binding and transfer of the packet about the pointer forwarding scheme, to recognize the movement in the PMIPv6 network environment, and to evaluate the performance of the proposed method. The performance shows it is better, as compared with the conventional method, in terms of overall cost.

The composition of this paper is as follows. Chapter II explains about related work, and chapter 3 explains the dual pointer forwarding scheme considering mobility. Chapter 4 explains the analytic model and numerical result, and the conclusion is in chapter 5.

## Related work

### Overview of PMIPv6

**Figure 2:**
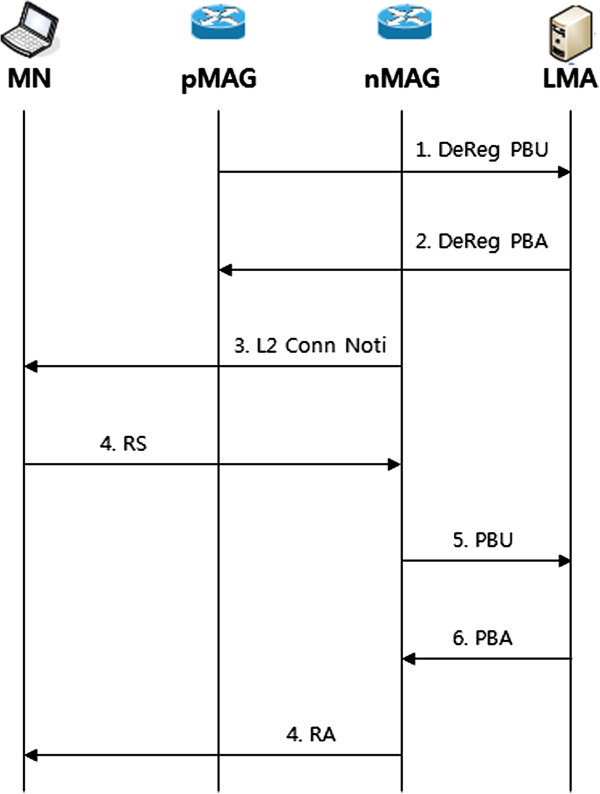
**PMIPv6 Handover procedure.**

PMIPv6 is based on MIPv6 as a protocol for providing a terminal network-based mobility. Figure 2 shows the handover procedure of PMIPv6.

If MN handover from pMAG to nMAG, pMAG inform the fact that MN is outside to LMA using DeReg. PBU(De-Registration PBU) message. LMA, having received the DeReg. PBU message that MN corresponding to pMAG sends (De-Registration PBA) DeReg. PBA, in response to deleting information about the MN corresponding to BCE to (Binding Cash Entry) has been deleted I announce. On the other hand, for the MN that is close to the range of their own, nMAG performs an authentication process in the AAA & Policy Store by using the MN-ID, and tries to connect to the nMAG, to put their MN-ID RS of (Router Solicitation) message nMAG. At this time, it contains information on how to set the address, and the address of the LMA to the MN service from the AAA & Policy Store and Home Network Prefix information of MN, such as the policy of the service. nMAG updates the current location of the terminal, by sending a Proxy Binding Update (PBU) message to MN that recognizes the policy profile of MN and MN-ID. At this time, if you do not search MN-ID in BCE, then LMA, having received the PBU, adds MN-ID into BCE. LMA sends a PBA (Proxy Binding Acknowledge) message to nMAG for terminal service. LMA using the address of nMAG is ready for the service, by using a bi-directional tunnel between nMAG and LMA. The nMAG sends a Router Advertisement (RA) message to the MN that LMA assigns MNP and IP address to the MN. The connection setup is completed, the nMAG sends traffic coming from the MN to LMA by using the tunnel connected to LMA, and sends traffic coming from outside to MAG, to manage the MN. However, the PMIPv6 support, only within a single domain, does not support inter domain mobility.

### Pointer forwarding scheme

The design of the pointer forwarding scheme has been proposed, to reduce the high costs of signaling in the cellular/PCS network environment. The proposed method can be avoided, by setting a simple pointer forwarded to the previous VLR, without registering the HLR and VLR of the new MN. When applied to a telephone, and the terminal, following the first command HLR requests, MN that has been first registered in the VLR connects the pointer forwarding chain of MN to the VLR current. These procedures reduce the HLR registration traffic, and are useful to the MN, if you want to change the registration location. The pointer forwarding method was introduced in the PCS network environment (Chen & Gu 2003; Ma & Fang 2003). The hybrid replication technique has been proposed in (Chen & Gu 2003), and a hybrid approach to determine the optimal replication per person and forward in the chain, can suggest, at least, the cost of location management. Whereas, in some way, in order to execute, the task management of location for all users, such as data structure and algorithms, can be executed. On the other hand, a two-level pointer forwarding structure was designed (Ma & Fang 2003). It can also be installed in the Mobility Agent (MA), to reduce the signaling traffic of location registration, by adding another step in the positional relationship between them. The two-level structure of pointer forwarding focuses not on mobility techniques for the PCS/cellular network environment, but on IP mobility protocol. In the case of IP mobility protocol, a number of mobility management techniques have been proposed. A dynamic hierarchical mobility management scheme has been proposed (Ma & Fang 2004). This method calculated via analytical models the optimal length, derived from the chain generated in the home registration. However, based on MIPv6 (Mobile IPv4), the proposed structure does not consider the impact of path optimization. In addition, by assuming the inter-packet arrival process, according to the exponential distribution, we propose a cost analysis model. However, the inter-packet arrival process does not follow an exponential distribution. A pointer forwarding scheme is proposed in the MIPv6 environment (Paxson & Floyd 1995; Chu & Weng 2002). The pointer forwarding scheme provides service for a single mobility domain, and introduces a new PFMA (Pointer Forwarding Mobility Agent) service. When the MN enters a new mobility domain, it sends a BU message to the previous PFMA. Therefore, it would be possible to reduce the number of binding updates. The proposed structure in MIPv6 is evaluated by using a simple analytical model. However, it shall not be calculated for the optimal forwarding pointer chain length. In addition, if mobility domain includes access router, PFMA and pointer forwarding occur frequently. In this case, the pointer-forwarding performance is certainly reduced. The pointer forwarding technique was applied in HMIPv6 networks (Yi & Hwang 2004). In this way, the pointer chain is set up between the access routers, instead of the MAP. Therefore, this approach does not reduce the binding update message, by handoff between the MAP. In this way, the inter-MAP handoff can’t reduce the binding update message to the HA. As mentioned before, handoff between MAP does not happen, if the domain size is enough. Therefore, the pointer forwarding scheme brings limited improvement in the MAP. Moreover, it does not evaluate the impact of route optimization, and the optimal length of the pointer chain.

### Applying pointer forwarding in the PMIPv6 network

PMIPv6 is designed to manage network-based mobility protocol, and does not perform a signal associated with the handover in MN and location registration procedure. Therefore, it has the advantage of being able to reduce the delay time of the mobility management, and the load of MN. However, all messages from PMIPv6 are basically transmitted via the LMA. MAG must be performed, to register the location to LMA every time the MN moves; these location registration procedures increase traffic to the LMA, and the overall traffic that is passed to the network. (The farther the distance of the LMA and MAG, the longer the location registration procedure for the delay is). In a proxy mobile IPv6 network environment, the pointer forwarding method is a way to reduce the cost of location registration, the registration procedure is omitted, and the MAG to the LMA in a remote location, by taking into account a large number of users, moves the MAG to be located in close proximity to the point forwarding (Yi et al. 2010). However, in this method, between the LMA and the LMA, MN has been considered for moving by being limited to one LMA, apply the transfer of pointers although moving highway vehicle and KTX (Korea Train eXpress) moving speed is fast no consideration of the mobile part is fast to a limit point is generated for transferring loads between a pointer that is incremented when receiving the package sent by the mobile. A state recognized pointer forwarding method is proposed in (Yan & Lee 2012), to recover the limitations of the above. The signaling overhead is mentioned in (Yi et al. 2010), but it considers the download and upload packets, according to the mobility of the MN. Due to the download and upload of mobility considerations, these apply, depending on the state of the MN. The state of MN use HI message (Internet control message protocol v6: ICMPv6) (Conta & Deering 2006). MN informs busy or idle status to MAG, using the HI message. If the state of the MN is Idle, the pointer chain is gradually increased in length; in the case of busy, MN initializes the length of the pointer chain, and updates the location registration procedure from LMA to MN (Yan & Lee 2012). In this method, the tunneling overhead is reduced, due to the packet transfer method of pointer forwarding, but it is possible to bring a performance decrease, as MN determines the state of busy and idle, so overhead MN is bringing increased battery usage this MN increase according to that you were to use the resources by overhead MN. In addition, for fast movement confined to the internal LMA, applying the forwarding pointer seemed to move fast movement speed KTX high-speed car did not consider the movement between the LMA and the LMA, MN consideration also has limitations (Yan & Lee 2012). Therefore, in this paper, due to the high speed development of transportation due to the domain move inside, and a large number of LMA, and considering the mobility between the present dual pointer forwarding signaling overhead, reducing the effect. In addition, due to the tunneling overhead and packet transmission, considering the mobility-aware binding update through the MAG and long-range CN (LMA), to manually register reduces the overhead of the tunneling.

## Cost-effective dual pointer forwarding-based handoff scheme

In this chapter, we present a binding update process based on a cost-effective dual pointer forwarding technique and packet transfer procedure, so proposing a mobility adaptive binding update procedure of CN.

### Binding update procedure

The handover between domains is not defined in PMIPv6, but the movement between domains is frequent, according to the development of transportation, and diversification into mobile devices. The mobility of Inter-domain causes high handover delay, packet loss and signaling overhead, according to the fast-moving of the MN. For this reason, two methods of a pointer forwarding scheme are proposed. First, when MN moves inter domain, make a pointer forwarding chain between the domains (LMA). The PMIPv6 handover between domains is not clearly defined. This is because of the not defined part of the information sharing of the home network prefix (HNP), when the MN moves from pLMA to nLMA. However, when MN enters from pLMA to nLMA area, nLMA, to run the connection (LMA of the CN another), based on the information of HNP received from pLMA, by running the binding update to request HNP information to pLMA, must perform a binding update. This is the point of the packet loss and handover delay, according to heavy signaling overhead occurring. So pointer forwarding between pLMA and nLMA is supposed to set, and LMA (the distance between pLMA and nLMA) is much shorter than sending a binding update to CN (LMA connected to MN). This can reduce the high signaling overhead of the binding update that occurs during the movement of the LMA in the PMIPv6 network environment. The pointer forwarding scheme maintains a pointer table (PT) per each LMA. Each PT consists of four fields: ID1, ID2, CURRENT. The ID1 field is composed of MN-HoA that is connected to the first, ID2 field is composed of MN-ID and CURRENT field uses LMAA (LMA address) of pLMA. But, the NEXT field is composed of LMAA (LMA address) of nLMA, to move next. For example, if the value of the NEXT field is NULL, then the value of CURRENT field is LMAA of LMA, so this means that MN stays in the CURRENT LMA.

**Figure 3:**
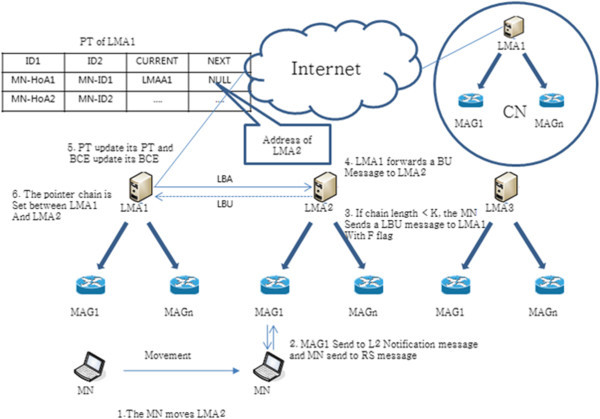
**PF scheme of an inter domain binding update.**

**Figure 4:**
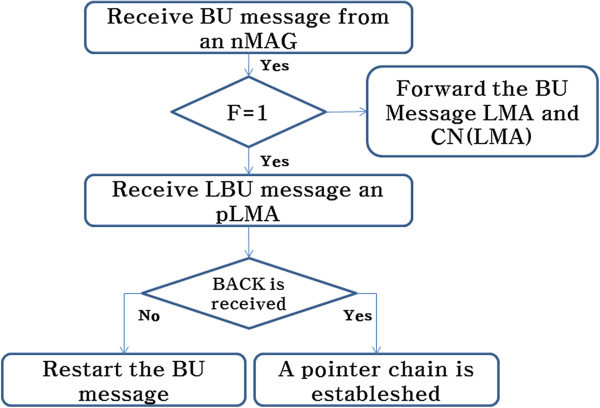
**LBU procedure between LMA.**

Figure 3 shows the binding update. Each value of CURRENT and NEXT is the LMA1 address (LMAA1) and NULL, respectively, after the first completed the initial registration of the LMA. If MN accesses a new LMA2, L2 connection information is sent to MN in MAG connected to LMA2, and MN sends an RS message (steps 1 and 2). The LMA2 received the request, is smaller than the K value of the pre-determined length of the link pointer forwarding chain, LMA2 transmits a LBU (LMA Binding Update) message to the LMA1. This message has the F flag values (steps 3 and 4). The LMA1 receiving LBU message updates PT (CURRENT: LMAA1, NEXT: LMAA2), then sends an LBA (LMA Binding Acknowledgement) message to LMA2 (Steps 5 and 6). LMA2, having received this message, increases the LBU message pointer chain variable L of LMA1 to 1, as shown in Figure 4, and sets the pointer chain. Binding update after that filled the value of each CURRENT and NEXT in PT of LMA1 with the LMA1 address (LMAA1) and LMA2 address (LMAA2), and removes MN information from BCE. Then, the LMA2 finishes the binding update, after filling each CURRENT and NEXT with the LMA2 address (LMAA2) and NULL, respectively.

Figure 4 shows the procedure for handling the LBU message. The LBU procedure, when the current length chain L is smaller than the length of chain K values being predefined, makes a chain to the next LMA. If you have the same values of L and K when the MN handovers, remove the chain and connect from the last LMA to CN (if L is greater than or equal to K, F > 1).

Second, when MN moves between MAGs within LMA, make a pointer forwarding chain. When MN moves from pMAG to nMAG for the PMIPv6 network environment, nMAG sends L2 connection information to MN. Receiving this message, MN sends an RS message to nMAG, and nMAG sends a proxy binding update (PBU) message to the LMA. LMA updates BCE if MN is present in the local BCE; if not, LMA sends a proxy binding acknowledgement (PBA) message to nMAG, after registration of a new MN. Then, LMA sends an RA message to MN requesting RS. LMA1 removes MN information from BCE, when MN is away from LMA1’s area. These procedures cause high signaling overhead on the inside of the LMA, and bring handoff latency. The pointer chain that is set between MAGs, is similar to the handover between the LMA. Pointer chain configuration is set between pMAG and nMAG, this is much shorter than the distance from MAG to LMA in the PMIPv6 network. Therefore, in the PIMPv6 network environment, the binding update message sent to (that are not sent to the LMA) pMAG is able to reduce the signaling overhead in the LMA within. Pointer forwarding scheme maintains PT on each MAG, and PT consists of the following four fields: ID1, ID2, CURRENT, NEXT. The ID1 field is composed of MN-HoA that is connected to the first, ID2 field is composed of MN-ID and CURRENT field uses pMAG of PCoA (Proxy Care-of-address). But, the NEXT field is composed of the PcoA of nMAG to move next. For example, if the value of the NEXT field is NULL, then the CURRENT field is the PCoA of MAG currently connected, which means that the MN has remained MAG CURRENT.

**Figure 5:**
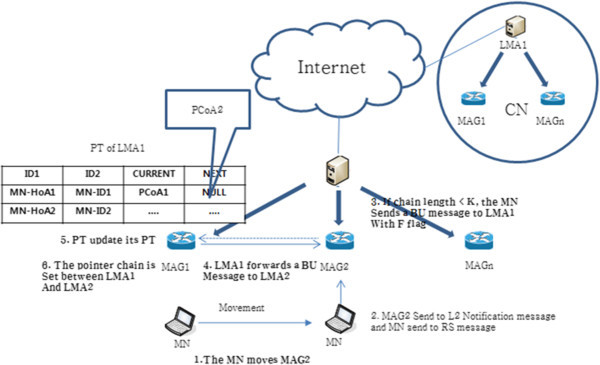
**Binding update within the domain of the PF scheme.**

Figure 5 shows the binding update procedure. When the initial registration is complete after that MAG is connected for the first time, the value of each of the MAG CURRENT and NEXT is PCoA of MAG1 and NULL respectively. If MN is connected to the new MAG2, MAG2 sends an L2 notification message to MN, and MN sends an RS message to MAG2 (step 1, step 2). If the length of the connection chain pointer forwarding is smaller than the predefined value of K, MAG2 sends a BU message to MAG1. This message has the value of the flag F (step 3, step 4). The MAG1 updates PT (CURRENT: PCoA1, NEXT: PCoA2) after receiving the BU message, and sends BA message to MAG2 (stop 5, stop 6).

**Figure 6:**
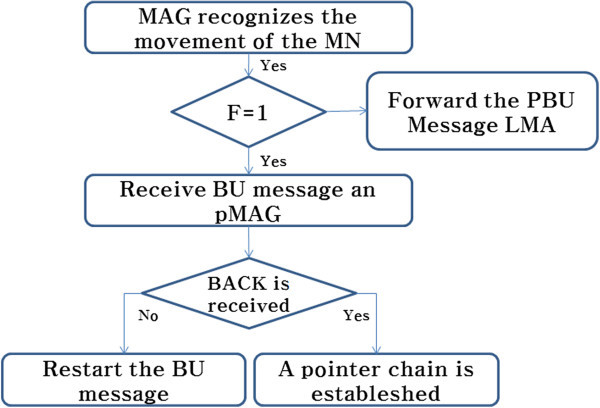
**The procedure of binding update from MAG.**

The BU message from the binding update procedure from MAG in Figure 6 is maintained, until L is smaller than the pre-determined value of K (the maximum length of the pointer chain). When K and L are equal in size, then the pointer chain is removed, and sends PBU to the domain LMA, and resets the pointer chain (L, K is greater than or equal, F > 1). The effect of the pointer forwarding scheme is to avoid excessive packet delay, so the performance evaluation of chapter IV clearly describes a standard PMIPv6 binding update, according to the size of the domain (LMA).

### Packet transmission procedure

The packet transmission procedure follows in accordance with the procedures of the pointer forwarding scheme. Packet transmission delivers using two pointer chains. The first packet transmission is delivery between domains at handover, the second is delivery within the domain, according to the MN to move.

**Figure 7:**
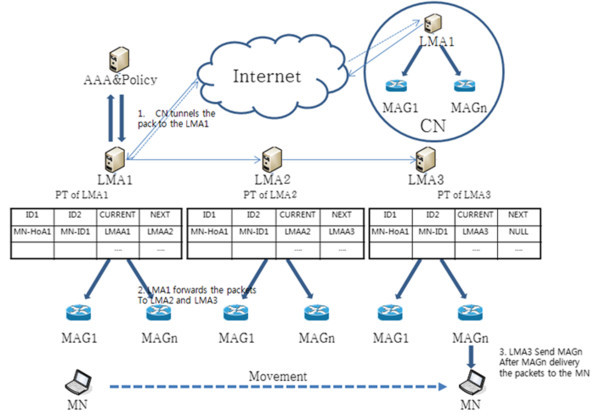
**Packet transmission between domains on pointer forwarding scheme.**

Figure 7 shows packet transmission between domains. Packet transmission, according to the MN between domains to move, shall be sent to the HA, when the CN (another LMA) first passes the packets to the MN (LMA itself has a Home Agent in PMIPv6). The LMA1 registers CN within LMA1 to HA (step 1). In the PMIPv6 network environment, LMA is responsible for the role of the HA, so LMA generates HNP by authentication to the AAA & Policy, instead of the HA registration process, in accordance with the MN moving. When the MN moves to LMA3, MN is connected to LMA3, but since the packet was connected to CN that has the LMAA (LMA address) of LMA1 to connect to first, the packet reception from CN is not LMA3, but LMA1. When LMA1 receives the packet search pointer table for packet transmission, packets are passed along the chain, until NEXT is NULL.

LMA1 sends packet to LMA2, since NEXT is the LMA2 address, not NULL, also LMA2 sends packet to LMA3, since NEXT is LMA3, not NULL (step 2). The NEXT value of point table in LMA3 is NULL (Moves the pointer chains at a time 1). LMA3 searches information about MN-ID of MN from BCE (Binding Cash Entry), because of nothing to deliver to the next LMA. The subsequent packet forwarding method follows the scheme of PMIPv6 (Step 3). MN accesses the LMA3 area, MAG sends L2 connection information to MN. MN try to connect to the MAG with a router solicitation (RS) message including MN-ID, and MAG performs the authentication process on AAA & Policy Store using the MN-ID. At this time, AAA & Policy Store has got the information of how to set the address and the address of the LMA to service in MN, Home Network Prefix information of MN, such as the policy of the service. MAG it recognizes and the MN Policy Profile and the MN-ID will be updated to the current position of the terminal, to send a PBU message to the LMA that is in charge of the MN. At this time, LMA, having received the PBU, adds MN-ID, if you do not search it. LMA for terminal services sends PBA message to the MAG. LMA, making a bi-directional tunnel between MAG and LMA, is ready for service, using the address of MAG. MAG sends a router advertisement (RA) message to the MN allocating an IP address, and HNP assigned by the LMA to the MN. When the connection has been set up, MAG sends to the MN the packets received through the pointer chain, and sends to the MN the packet transmitted from the LMA3.

**Figure 8:**
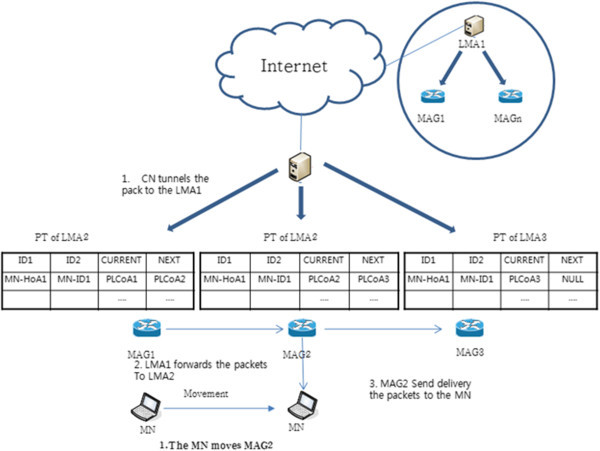
**Packet transmission within domain on pointer forwarding scheme.**

Figure 8 shows packet transmission within domain on the pointer forwarding scheme. Pointer forwarding in the way of packet transmission is as follows. CN sends to the LMA packet of the first time it sent the packet to the MN from CN (another LMA). CN sends the packet through the tunnel by LMAA (step 1). PCoA in the pointer forwarding scheme, registered in the last is not a PCoA that is used in MN. For example, PCoA the most recently registered in LMA is PCoA1. On the other hand, the current PCoA is PCoA3. The MAG1 so as the transmission of packets to the MN should be re-routed. Rerouting check NEXT field from PT of MAG where the most recently registered on the LMA and must determine whether the MN is or not.

MAG1 sends packet to the MN when the NEXT field on PT is NULL. In other words, MAG1 sends a packet to MAG2 when the NEXT field on PT is not NULL (step 2). After forwarding to the MAG the amount of the pointer chain length, MAG of which the NEXT field is NULL sends a packet to MN (Step 3). MAG2 of which the NEXT field is NULL sends a packet to MN in Figure 8.

**Figure 9:**
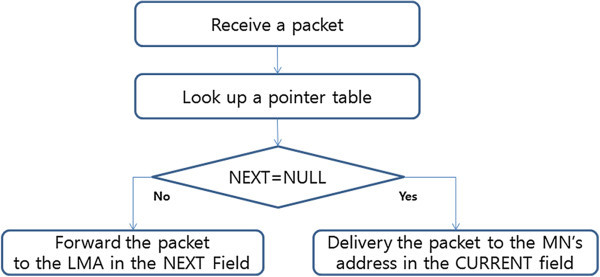
**The movement procedure of the pointer chain.**

Figure 9 shows the movement procedure of the pointer chain. Repeated in the formation of MN in the pointer forwarding scheme, repeated to Node (LMA or MAG) re-entering a previously visited. Thus, the configuration of the node list (LMA or MAG) visited already should be avoided. If you belong to the node list of the new Node being visited, it means forming a loop. MN then follows the variable L, the length of the pointer chain. If the new node becomes i-th in the node list, the variable length of the pointer has a value of I. At the same time, the MN sends a BU message to the current node. A BU message in response to the current Node retrieves and updates the iteration. PT registers entries (MN, LCoA, NULL). The pointer forwarding scheme is applied by two. Where in the domain maintains only a pointer chain between the domains, and moved to a different domain, and proceed with the initialization in Within PMIPv6 domain, after when the MN moves within a domain in a domain, set the pointer chain between the MAG and the MAG.

### Mobility adaptive PF scheme (mPF)

**Figure 10:**
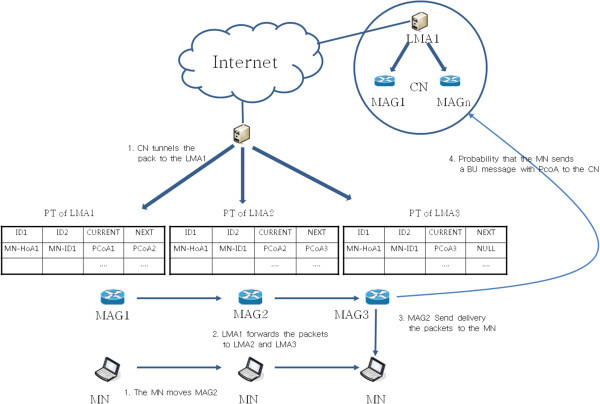
**Packet transmission procedure on mPF scheme.**

If you apply mPF for the PMIPv6 network environment, between the CN (another LMA) and LMA, and LMA and MAG, BU signaling overhead and tunneling overhead can be reduced. Tunneling for PBU between the MAG and the LMA is required; but with the signaling for that, there is a possibility of causing overhead within the LMA. This is the motivation of the binding update considering mobility. MN can be classified into two types in MBU. The classification can be done by measuring the time interval between the router advertisement (RA) messages received from the other MAG. When T1 is the waiting time for the RA message from MAG and moving, and waiting, T2 is the RA from another MAG to move and the waiting time, the MN subnet residence time (t2-t1) can be explained. MN enters a new subnet of the MAG, and it is possible to estimate the residence time in the new MAG subnet, using the residence time of the subnets that are previously measured. Evaluation in the exponentially weighted moving average (EWMA) technique to mitigate the effects of a change in the measured residence time can be utilized. After that, the MN compares the expected residence time to a pre-defined threshold δ. If the expected value is smaller than δ, the MN is considered to be the fastest; if not, it is considered to be slow. PLCoA is the percentage of direct notifications to CN from the MAG. Therefore, while the MN is slow, and is then notified to the CN, the fast MN sends a BU to the LMA. Therefore, the mPF scheme includes the dual pointer forwarding mentioned earlier, and mobility awareness through direct notification between the CN and the MAG, and the MAG and LMA, and the tunnel between the LMA and the CN signaling overhead and packet transmission overhead are reduced. The numerical analysis for the calculation of PLCoA is in Chapter IV. Thus the fast MN in the mPF scheme using LMA sends BU in order to reduce the binding update traffic and at the same time, the slow MN can reduce the unnecessary LMA processing cost of packet forwarding procedure. Figure 10 describes the procedure of packet transmission in the mPF scheme.

The transmission of packets in the mPF schemes is as follows. In the case of the mPF scheme, the PCoA is transferred to CN by the ratio of PLCoA. mPF sends a packet in the same way as the pointer forwarding scheme, as follows If the MN is fast, CN (another LMA) sends packets to the MN, when the first packet is sent to the LMA. The CN delivers packets using LMAA and tunneling (Step 1). The PCoA that was recently registered is not a PCoA that is used in the MN. For example, the PCoA that is the most recently registered to the LMA is PCoA1. On the other hand, the current PCoA is PCoA3. The MAG1 So the transmission of packets to the MN should be re-routed. When the re-routing checks the NEXT field of the PT from the most recently registered in the LMA, you should check whether the MN is (NEXT is null, MAG includes MN). If the NEXT field on PT is NULL, send packets to the MN. In other words, because MAG1 has a non-NULL value in their NEXT field of PT, it forwards the packet to the MAG2, and sends a packet to MAG3 because of the presence of NEXT (step 2). NEXT and then forwarded to the MAG as a pointer chain length after passing, MAG having NEXT field is NULL sends packet to the MN (Step 3). MAG2 in Figure 10 has a NULL for the NEXT field. MAG forwards the packet to the MN. However, if the MN is slow, CN becomes able to send packets directly to the MAG3 through the registration process, by sending a PCoA directly to the CN, without passing through the LMA (Step 4).

## Performance evaluation

In this chapter, we consider the binding update and packet transmission through improved analytical models, to quantify the total cost. The total cost is analyzed between the domains, and within the domain, by each scheme of PMIPv6, PF, mPF. Unlike (Ma & Fang 2004), the binding update and packet transmission time interval are defined by the last data packet from the first data packet (Zhang et al. 2002). In this paper, we have modeled the following notation for analysis.

### Cost modeling

Abbreviations and symbols for formal notation of cost analysis.

- *E*(*L*_*s*_): Average session length (number of packets)
- *E*(*N*_*D*_): The arrival time inter average cross-domain session
- *E*(*N*_*C*_): The arrival time inter average cell cross-session
- *B*_*F–LMA*_: unit pointer installation costs(between LMA and LMA)
- *B*_*F–MAG*_: unit pointer installation costs (between MAG and MAG)
- *B*_*LMA*_: PBU cost per unit from MAG to LMA
- *P*_*F–LMA*_: The cost of from previous LMA to next LMA
- *P*_*F–MAG*_: The cost of from previous MAG to next MAG
- *C*_*LMA–LMA*_: The number of hops between LMA and LMA.
- *C*_*MAG–LMA*_: The number of hops between LMA and MAG
- *C*_*MAG–MAG*_: The number of hops between MAG and MAG
- *w*: The total number of data session packet before routing optimization
- *P D*^*PMIPv6*^: The packet delivery costs of from CN to LMA
- *P*_*LCoA*_: The rate of sending message from MAG to CN (MAG) directly

#### PMIPv6

In the PMIPv6 network environment, BU messages being generated in the within domain occur when the MN moves between the MAG. When the MN moves from pMAG to nMAG, it sends a PBU message to the LMA.

In the PMIPv6 network environment, the packet delivery cost that occurs in the within domain, is the cost of packet delivery from LMA to CN, and the cost of packet delivery from MAG to LMA. The packet delivery costs are as follows.

In the PMIPv6 network environment, a BU message occurs when the MN moves between domains (between LMA). (In the case of PMIPv6, BU occurs only in the CN, because LMA contains HA). The cost of BU from LMA to CN (another LMA), and cost of PBU from MAG to LMA occurs. The cost of BU in PMIPv6 is as follows.

In the PMIPv6 network environment, when the MN moves to between domains (between LMA), packet delivery is a packet forwarding cost to MN from the MAG of nLMA to the MN. The costs of packet delivery are as follows.

#### Pointer forwarding (PF)

When the MN moves within the domain in the case of the pointer forwarding scheme, it sends a BU message to the LMA connected to MAG, and MAG performs initialization processing of the PMIPv6 connection. MAG sends a BU message to the LMA, when the first packet arrives. In the other case, when the pointer chain value reaches a predefined value K, a BU message is sent to LMA. If the MN has moved between the MAG, but if the length of the chain of pointers within MAG is less than K, the BU message will be sent to the previous MAG.  represents the average number of cross-cell within the ith LMA domain.  represents the pointer reconfiguration, and  represents pointer updates within the ith domain. So the way of cost calculation in the pointer forwarding scheme is shown below.

The pointer forwarding scheme within a domain can reduce the cost of BU, but the cost of packet transmission is increased. The reason for this is due to packet transmission using the pointer chain. After reconfiguration of the pointer chain, all of the K use an approximation for the assumed movement to be K/2, the average length of the pointer chain crossing the MAG. The way of packet delivery cost calculation in the pointer forwarding scheme is shown below.

When the MN moves within the domain in the case of the pointer forwarding scheme, it sends a BU message to CN (another LMA) being connected to the domain (LMA), and the MN proceeds to initialization processing of the PMIPv6 connection within the domain. The MN sends a BU message to the CN (another LMA), when the first packet arrives in the domain (LMA). In other cases, the LMA, when it reaches a predefined pointer chain value K, sends the BU message to the CN. If MN has to move between domains, but if the length of the pointer chain is less than K in LMA, it sends a BU message to the previous domain (LMA). represents the average number of the domain crosses between the i’th LMA domain, and  the pointer reconfiguration, and  updates the pointer in the ith domain. After that, the calculation of BU cost in the pointer forwarding scheme is as follows.

The pointer forwarding scheme reduces the BU cost when moving between domains, but the cost of packet transmission increases. The reason is that packet transmission uses a pointer chain. After pointer chain reconfiguration, the average length of the pointer chain that crosses the LMA for all K, is K/2 approximate values for homes on the go. The cost of the packet delivery in the pointer forwarding scheme is as follows.

#### Mobility adaptive PF scheme (mPF)

In mPF, it is possible to receive PLCoA from CN in mPF. It is possible to calculate the lifetime *T*_*BU*_ that all the PcoA do binding updates across to MAG, considering the MN move slow. The cross ratio (*μc*) of the MN’s MAG should be considered, to calculate the additional cost of BU. Then the average number of crosses to the subnet during *T*_*BU*_ is *μcT*_*BU*_. Therefore the cost of BU in the mPF scheme is as follows.

If the mPF scheme is applied to the pointer forwarding scheme, then the cost path of pointer forwarding is that MAG notifies direct to CN (another LMA), according to the packet time received from LMA. When MAG informs PCoA to CN, the CN receiving PCoA does packet delivery to MAG. It’s the same as PMIPv6. Therefore, when MBU is applied to the packet delivery, the cost of PF is as follows.

In the mPF environment, the difference of inter domains and within domain is the same as the difference of pointer forwarding scheme. mPF is the inter domain PF between LMA. In the PMIPv6 environment, the cost of mPF BU between domains is shown below.

In the mPF environment, the cost of packet delivery is the same between domains and within domain. The packet delivery cost of mPF between domains is as follows.

### Numerical analysis of the results

Table 1 shows the values of the parameters that were used. The value of the MA and the value of applying the MA process costs are the value (Xie & Akyildiz 2002) of hops. SMR (session-to-mobility) = *λs*/*μc* is defined for mobility effect analysis. *λs* is the session arrival rate, and *μc* is the subnet cross ratio. Similarly, if the exponential distribution is set to the session arrival rate, and the subnet run residence time at (Xiao et al. 2004) is 1/*λs* and 1/*μc*, the average number *E*(*Nc*) to cross a number of MAG/LMA per session is *μc*/*λs*. This means the LMA and domain cross ratio, and the number of MAG within the domain. Specifically, the *μ*_*D*_ is an approximation of , and n is the number of MAG within the domain (Wang & Huey 1999). Therefore, when described as an exponential distribution, the average residence time of the LMA cross ratio of the MAG domain per session, 1/*μ*_*D*_ and *E*(*N*_*D*_) is the same as . In the above analysis, N is 49 shall be determined by (Zhang et al. 2002). The subnet residence time *t*_*c*_ is a random variable. Then, *P*_*LCoA*_ can be obtained as follows.

**Table 1:** **The parameter values for numerical analysis**

Parameter	Value
*B*_*F*-*LMA*_	2
*B*_*F–MAG*_	1
*B*_*LMA*_	4
*P*_*F–LMA*_	3
*P*_*F–MAG*_	1.5
*C*_*LMA–LMA*_	8
*C*_*MAG–LMA*_	2
*C*_*MAG–MAG*_	1
*PD* ^*PMIP*^	15
*T*_*BU*_(*S*)	180

*δ* is a pre-specified threshold.

#### The length of optimized pointer chain

**Figure 11:**
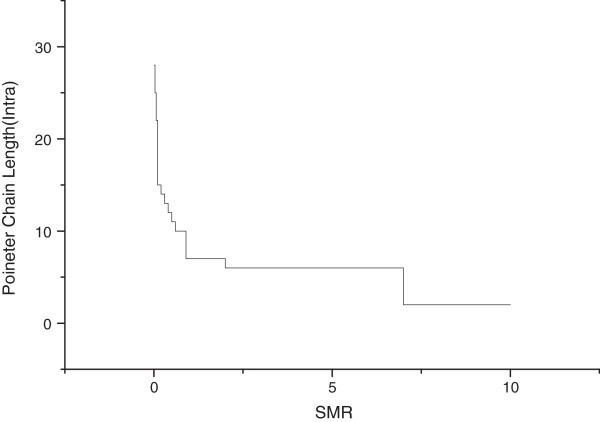
**SMR within domain vs pointer chain length.**

**Figure 12:**
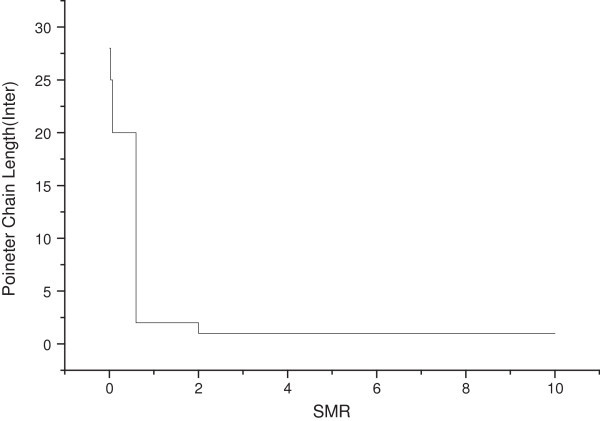
**SMR inter domain vs pointer chain length.**

Figures 10 and 11 show the influence of the SMR on the optimal length of the pointer chain. The case of Figure 11 represents the optimal length of the pointer chain within the domain, and Figure 12 represents the optimal length of the pointer chain in the inter domain (inter LMA).

Figure 11 shows the optimal pointer chain length according to the SMR within a single domain. The pointer chain is generated continuously, in accordance with the movement of the MN between pMAG and nMAG, and Figure 11 shows the impact of SMR on the generated pointer chain at this time. If the SMR is low, and there is a lot of MN mobility, the mobility is more important than the arrival rate of the session. Therefore, to reduce the cost of BU is more effective, than reducing the cost of packet delivery. The length of the pointer chain should be longer, in order to reduce the BU. The reason is that the cost to request BU from nMAG to pMAG is very low, compared to the cost of requesting BU from MAG to LAM. But if the value of the SMR increases, the MN’s mobility is relatively small, compared to the session arrival rate. For such a situation, the cost of packet delivery per total cost is major, and a short pointer chain is favorable to reduce the overhead of packet delivery. Therefore, when SMR is small, then the length of pointer chain within domain (inter MAG) is time to increase; when SMR is big, then the length of pointer chain is time to decrease.

Figure 12 shows the optimal pointer chain length according to the SMR, when the MN moves to inter domain. The case of mobility within domain and inter domain is not significantly different. If the value of SMR is small, then inter domain mobility is frequent, and in this case, the MN’s mobility is more important than the arrival rate. So, to reduce the cost of BU is more effective than the cost of the BU. The cost of BU is to send connection information to the previous domain (pLMA), from the moved domain (nLMA). It is more cost effective to set the pointer chain in the previous domain (pLMA) from the moved domains (nLMA), rather than to proceed with BU connecting to CN. If SMR may be relatively smaller within the domain, the domain moves between the MN moves within the domain, because the session arrival rate becomes more important than the mobility. In this situation, it is more effective to reduce the packet delivery overhead, because the cost of packet delivery is large, compared to the cost of BU. Thus, the optimal length of the pointer chain decreases with an increase of SMR.

#### The effect of SMR

**Figure 13:**
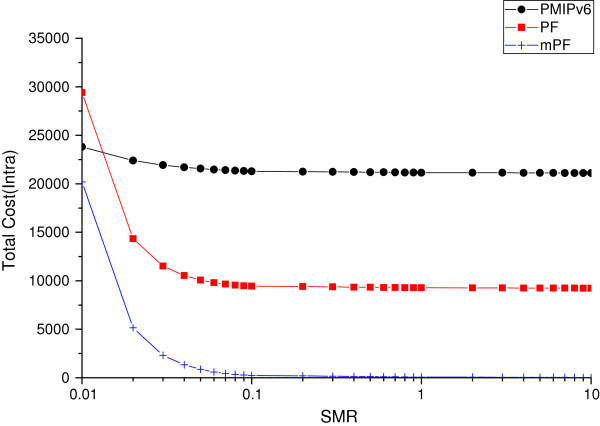
**The impact of the total cost of SMR within domain.**

**Figure 14:**
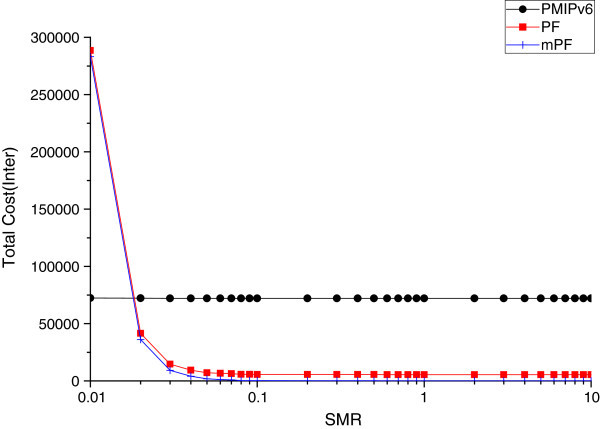
**The impact of total cost of SMR between domains.**

The total cost due to SMR is variable. Figure 13 and Figure 14 shows the effect of the total cost of PMIPv6, PF, and mPF, due to change in the SMR.

Figure 13 shows that the total cost of mPF is most effective when the MN moves within the domain, according to the change of SMR value. By comparing the total cost of PMIPv6, PF, and mPF, according to the change of SMR value, if SMR is less than 0.02, then the total cost of PF compared to PMIPv6 is high, and the total cost of mPF shows the performance much better than that of PF and PMIPv6. When SMR is small (SMR 0.02 or less), mobility is high, the performance of PF shows a low level of 20%, due to the signaling to form a pointer chain, and the performance of mPF shows 20% higher than PMIPv6. But if SMR is greater than 0.02, the performance of both PF and mPF shows a higher performance than PMIPv6, and the cost of PF and mPF show more than 60% and 90%, respectively, based on the SMR 0.1.

Figure 14 shows the effect of total cost according to the SMR change, when MN moves between domains. If the mobility is very fast, in the case of 0.02 or less of SMR value, the total cost shows the minimum cost in the PMIPv6 network environment; but in the case of 0.02 of SMR value, the performance of PF and mPF is much higher; and in the case of 0.08 of SMR value, the performance is very low. If you do not move multiple domains in a very short time, it shows that the PF and the mPF are more effective. In the case of PMIPv6, SMR shows that the result is not relatively affected by the mobility and packet arrival rate, but generally it shows a much higher level of total costs, compared to the PF and mPF.

#### The effect of session length

**Figure 15:**
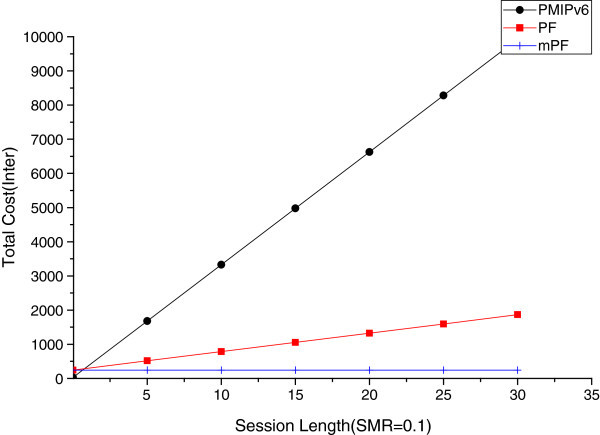
**The effect of session between session (SMR = 0.1).**

**Figure 16:**
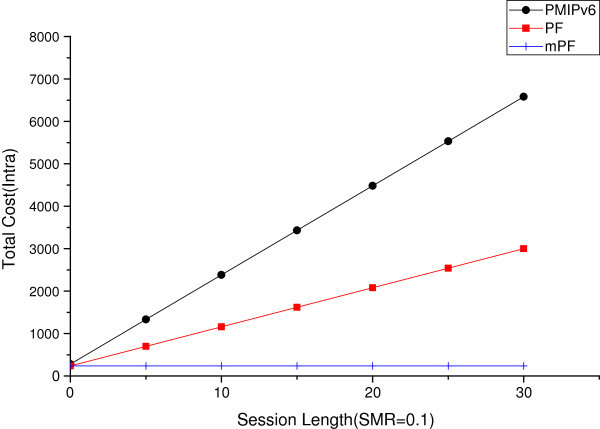
**The effect of session length within domain (SMR = 0.1).**

**Figure 17:**
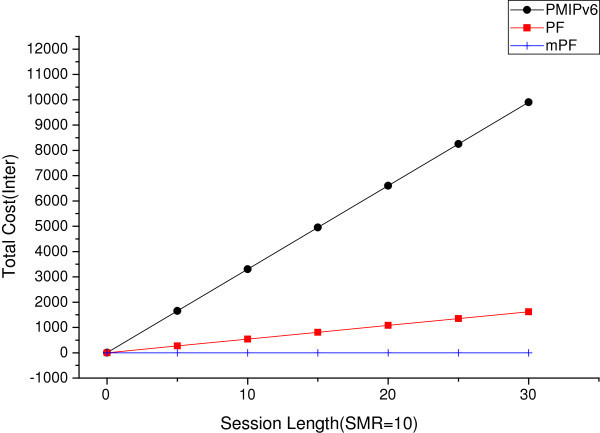
**The effect of session length between domains (SMR = 10).**

**Figure 18:**
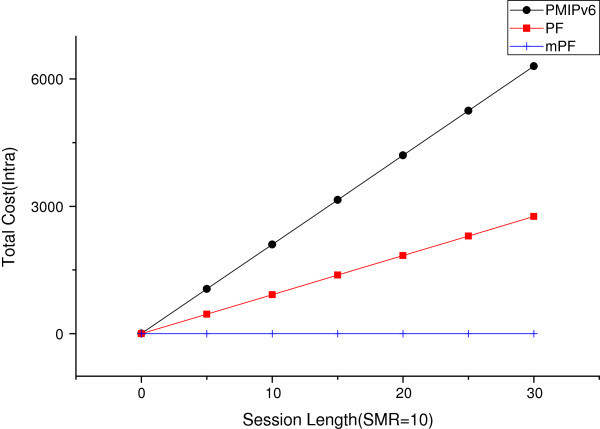
**The effect of session length within domain (SMR = 10).**

It is time to analyze the effect on the length of the session between the domains and within domain, and in cases of the size of SMR being small and big (SMR = 0.1, SMR = 10). Figures 15 and 16 shows the total cost of the session in the case of the value of SMR 0.1, and Figures 17 and 18 show in the case of the value of SMR 10.

Figure 15 shows the variation of the total cost of SMR value of 0.1, when the MN moves between domains, in the case of the session length increases. In the case of PMIPv6, BU occurs continuously from domain (LMA) to CN (another LMA), and the cost is much higher than in the PF and mPF. The mobility is a more important factor than the session arrival rate, because mobility is high, so the value of SMR is 0.1. And the impact of cost reduction of BU is greater than reducing mobility. But, in case of PMIPv6, BU to CN occur repeatedly due to greater mobility, as shown vertically in Figure 14, as the total cost increases. In the case of PF and mPF, the information of a pointer chain when you move between domains, the non-BU CN previous domain (pLMA) and following domains (nLMA) between BU, so the total cost will increase.

Figure 16 shows the total cost of the SMR 0.1, when the session length increases within the domain. PMIPv6 mobility is a more important factor than the session arrival rate i.e. SMR = 0.1, when the MN moves from MAG to the domain (LMA). PBU continues to happen, and it seems that the total cost of PMIPv6 grows vertically. In the case of PF, you can see that the modest increase in overall costs over PMIPv6, because of performing BU for the pointer chain from pMAG to nMAG. The reason is that the cost of the pointer chain from pMAG to nLAG is much less than PBU from MAG to the domain (LMA). On the other hand, mPF shows a nearly constant value. However, when the length of the session increases, due to the session arrival rate being lower, BU using *P*_*LCoA*_ in CN, the total cost of mPF hardly increases, due to direct connection from CN.

Figure 17 shows the total cost due to an increase in the length of the session, when moving between domains in the SMR 10. When the value of SMR is 10, the session arrival rate is more important than the mobility. In this case, it is more important to reduce the cost of packet delivery, than of BU. In PMIPv6, it can be seen that the width of increase is reduced, but the total cost increase is more vertical than when the mobility increases. It is a more modest increase in the case of PF. But in the case of mPF, notifications to CN (another LMA) are close to a probability of 1. Thus, in the case of mPF, direct communication of MAG connected to MN and CN, the value is not nearly increased.

Figure 18 shows the total cost of the SMR 10. When the session length increases within the domain. Then in the case of the value of SMR 10, the session arrival rate is a more important factor than mobility. PMIPv6 than when the mobility increases, it can be seen that the total cost increase vertical increase the width of the line. PF more modestly increases. However, if the mPF’s inform the CN of the probability of PLCoA close to 1. Therefore, in the case of mPF and MN connected through the connection of the MAG and CN, the packet delivery value is almost not increased.

#### The effect of domain size

The size of LMA is n (the number of MAG in LMA). Two points of view affect the total cost. The first, LMA within the MAG is the  cross. MN and MAG, and is proportional to the number of hops between the LMA and the MAG and the BU costs of the LMA, depending on the size of the LMA. The hops log_*β*_(*n*) between the MN and LMA are the maximum number in the hierarchical structure. Therefore the BU cost in LMA is proportional to log_*β*_(*n*).

**Figure 19:**
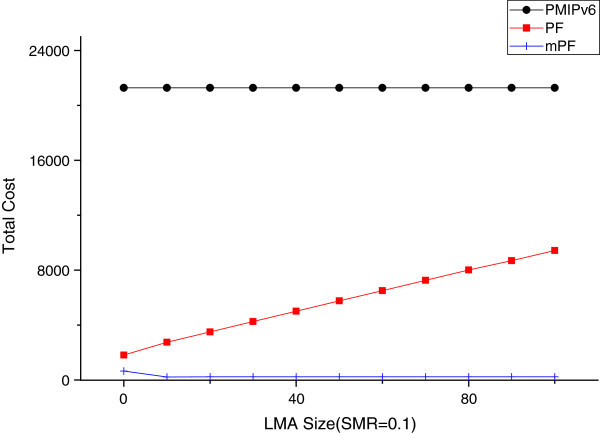
**The effect due to LMA size (SMR = 0.1).**

Figure 19 shows the SMR value of 0.1, when the impact on the domain (LMA) in the MAG can. In the case of mPF, the total cost is reduced relatively due to the size of the domain increases. So, mPF is more effective when the domain size is large. In the PMIPv6, it does not affect the size of the domain (LMA). But the relatively total cost of PMIPv6 is significantly large, compared to PF and mPF. Therefore, the difference is obvious between PMIPv6 PF and mPF. The BU cost is proportional to the size of the domain (LMA). When you send PBU within the domain (LMA), it increases, due to the number of hops between the MAG and domain. Therefore, PF and mPF reduce the BU between the domain and MAG, using a pointer chain to reduce the PBU in a large domain. PF and mPF show a better performance.

**Figure 20:**
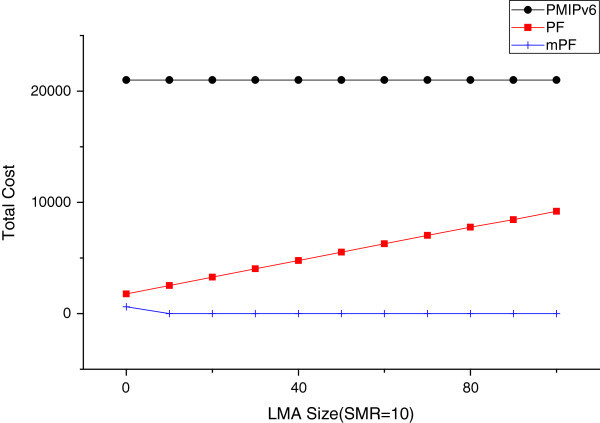
**The effect due to LMA size (SMR = 10).**

Figure 20 shows the impact of the number of MAG within a domain, in the case of the value of the SMR 10. In the case of mPF, the total cost decreases relatively, when increasing the size of the domain (LMA). mPF is more effective, when the domain size is large. PMIPv6 has little effect on the size of the domain. But the total cost of PMIPv6 is much larger relatively, compared to the PF and mPF. Therefore, the difference between PMIPv6, PF, and mPF is obvious. The reason is that the packet delivery costs in proportion to the size of the domain in the domain, due to increases in the inter domain, when the number of hops between MAG and domain (LMA). PF and mPF show a better performance.

## Conclusion

This paper proposes BU and PF (DPF) methods that consider the mobility in PMIPv6 networks. The proposed dual pointer forwarding scheme can decrease the cost of BU, and solve the high signaling overhead problem generated in LMA. The dual pointer forwarding scheme is proposed with two methods in this paper. The first is the pointer chain connection method between domains (LMA), which restricts movement between domains in PMIPv6. The second is proposed to reduce the high signaling overhead and packet delivery delays within domain that are generated by the pointer chain connection between MAGs within domain, when MNs move within the domain (LMA). The PF scheme between LMA reduces high signaling overhead, due to when the MNs move between domains. A mobility adaptive mPF scheme is used, where the length of pointer chain becomes long, or packet delivery delays occur with simultaneous signaling overhead between LMA and MAG. When the delay of response from LMA reaches a scheduled time, the packet overhead that is delivered from MN is decreased, due to registering PLCoA to MAG, where CN belongs to. Numerical analytic result is presented in this paper, after analyzing the effect of session and domain (LMA) size with between domains, and within domain, respectively. In addition, the effect from the optimal pointer chain length and SMR is analyzed. Finally, the proposed mPF scheme shows that it is excellent in performance, and reduces the total cost by comparing mPF to PMIPv6 and PF.
